# Impact of initial chemotherapy cycles and clinical characteristics on outcomes for HIV-associated diffuse large B cell lymphoma patients: The Central and Western China AIDS Lymphoma League 001 study (CALL-001 study)

**DOI:** 10.3389/fimmu.2023.1153790

**Published:** 2023-03-29

**Authors:** Chaoyu Wang, Yan Wu, Jun Liu, Haiyan Min, Yunhong Huang, Guo Wei, Wei Zhang, Min Wang, Xiaoqiong Tang, Hui Zhou, Yaokai Chen, Yao Liu

**Affiliations:** ^1^ Department of Hematology-Oncology, Chongqing Key Laboratory of Translational Research for Cancer Metastasis and Individualized Treatment, Chongqing University Cancer Hospital, Chongqing, China; ^2^ Henan Infectious Disease Hospital, The Sixth People’s Hospital of Zhengzhou, Zhengzhou, Henan, China; ^3^ Department of Hematology, Kunming Third People’s Hospital, Kunming, Yunnan, China; ^4^ Infectious Disease Department, Yunnan Provincial Hospital of Infectious Diseases, Kunming, Yunnan, China; ^5^ Affiliated Hospital of Guizhou Medical University, Guiyang, Guizhou, China; ^6^ Oncology Surgery, Public Health Clinical Center of Chengdu, Chengdu, Sichuan, China; ^7^ Peking Union Medical College Hospital, Chinese Academy of Medical Sciences (CAMS) & Peking Union Medical College, Beijing, China; ^8^ Department of Infection and Immunology, The First Hospital of Changsha, Changsha, Hunan, China; ^9^ The First Affiliated Hospital of Chongqing Medical University, Chongqing, China; ^10^ The Affiliated Tumor Hospital of Xiangya Medical School, Central South University, Changsha, Hunan, China; ^11^ Department of Infectious Medicine, Chongqing Public Health Medical Center, Chongqing, China

**Keywords:** HIV, DLBCL, prognosis, chemotherapy cycles, CALL

## Abstract

**Introduction:**

Little is known about the first line induction chemotherapy cycles for HIV-associated diffuse large B-cell lymphoma (DLBCL) as these are less common than HIV-negative lymphoma. Currently, the optimal treatment cycles option remains undefined. Therefore, we performed a multi-center study to analyze the clinical characteristics and outcomes of HIV-associated DLBCL patients in different treatment modes in China.

**Methods:**

Totally 273 newly diagnosed HIV-associated DLBCL patients at eleven large academic centers from October 2008 to October 2021, were analyzed.

**Results:**

In the entire cohort, the median age was 47 years (range, 21-90) at lymphoma diagnosis, and 223 patients were male (81.7%). One hundred and ninety-four (71.1%) patients were germinal center B-cell-like lymphoma (GCB) subtype. Most patients (65.2%, 178/273) had elevated lactate dehydrogenase (LDH), and advanced Ann Arbor stage (78.9% 213/273) at diagnosis. High international prognostic index (IPI) score (3-5) at diagnosis was found in 65.2% (178/273) of patients. One hundred and fifty-five patients (56.8%) had extranodal involvement. The median CD4 cell count was 168/μl (range, 2-1067), of whom 174 (63.7%) had a CD4 cell count below 200/μl. The median follow‐up of our cohort was 10.1 (0.1-160) months. The overall 2-year OS rates 58.0%. Median OS times in the 0, 1-3, 4-6, and >6 cycles chemotherapy cohort were 7.1 months, 20.0 months, not reached, and not reached, respectively (Hazard Ratio (HR)=0.549, 95% Confidence interval (CI) 0.451-0.667; *p*<0.001). Cox multivariate analysis showed that age ≥60 (HR=2.207, 95%CI 1.321-3.690; *p=*0.003), high IPI score (3-5) (HR=2.926, 95% CI 1.716-4.988; *p<*0.001), B symptoms (HR=1.928, 95%CI 1.192-3.119; *p=*0.007), elevated LDH (HR=1.696, 95%CI 1.031-2.791; *p*=0.038) and received less than 4 cycles chemotherapy (HR=0.520, 95%CI 0.424-0.637; *p<*0.001) were independent risk factor for adverse prognosis based on overall survival (OS).

**Discussion:**

These results demonstrated that 4-6 cycles chemotherapy were significantly associated with improved outcomes in HIV-associated DLBCL patients. However, >6 cycles chemotherapy did not further improve the survival of patients.

## Introduction

Human immunodeficiency virus (HIV)-associated lymphoma is a rare aggressive non-Hodgkin lymphoma. The incidence rate reported in 2017 was 100/100, 000 to 300/100,000 in population infected with HIV, becoming the highest incidence rate among HIV-associated cancers in the United States (1, 2). Diffuse large B-cell lymphoma (DLBCL) is the most common subtypes of HIV-associated lymphoma (3).

Median survival times only 5 to 8 months prior to the advent of combination antiretroviral therapy (cART), regardless of chemotherapy regimen or dose intensity for HIV-associated DLBCL patients. This is because patients are easily complicated by opportunistic infections, resulting in an increase in mortality (4, 5). With the wide application of cART, the HIV viral loads maintained good control and CD4 cell counts remain at a high level in HIV-associated DLBCL patients. So, these patients are no longer associated with severe infections. At the same time, these patients can receive dose intensity chemotherapy. Since the introduction of cART combined with intensity chemotherapy, studies have suggested that the overall outcome of patients with HIV-associated DLBCL has significantly improved over the past decades (6, 7).

In our previous single-center retrospective study included 86 HIV-associated aggressive B-cell non-Hodgkin’s lymphoma patients, of which 63 were HIV-associated DLBCL from July 2008 to August 2021. 22 patients (25.6%) received no anti-lymphoma chemotherapy because of fear of discrimination and poor financial situation. Median survival times in the patients who no received any anti-lymphoma chemotherapy was only 3.5 months, which was significantly lower than that of patients receiving chemotherapy (*p<*0.001) (8).

The optimal treatment cycles option of first line induction chemotherapy for HIV-associated DLBCL remains undefined (1). In view of the particularity of the patient population, it is difficult to accept long-term hospitalization chemotherapy, whether there is an appropriate reduction in the number of chemotherapy cycles, the survival of patients will not be affected? In this study, we aimed to report the real-world survival of HIV-associated DLBCL based on chemotherapy cycles at eleven large academic centers, as the largest cohort reported in China to date.

## Patients and methods

### Patients

All consecutive HIV-infected patients diagnosed with DLBCL between July 2008 and October 2021 were reviewed from eleven academic medical centers in China. This study was approved by each center’s Institutional Review Board and conducted according to the Declaration of Helsinki prior to data collection and sharing. The diagnosis was based on 2008 World Health Organization (WHO) classification criteria.

### Treatment and supportive care

cART included two nucleoside reverse transcriptase inhibitors and one nonnucleoside reverse transcriptase inhibitor. The chemotherapy cohort included patients who received any of the following regimens: etoposide, doxorubicin, vincristine, cyclophosphamide and prednisone (EPOCH); rituximab, etoposide, doxorubicin, vincristine, cyclophosphamide and prednisone (R-EPOCH).

### Response assessment

^18^F-fluorodexyglucose positron emission tomography/computed tomography (PET/CT) or computed tomography (CT) were performed for radiological evaluation. Responses were classified according to standard criteria per each institution, typically aligning with Lugano criteria, which including complete response (CR), partial response (PR), stable disease (SD) and progressive disease (PD).

### Statistical analysis

Progression-free survival (PFS) was defined as the time from lymphoma diagnosis to disease progression, relapse or death from any cause. Overall survival (OS) was defined as the time from lymphoma diagnosis to last follow-up or death from any cause. Survival was estimated using Kaplan–Meier curves and compared by the log-rank test. The Cox proportional hazards regression model was used in multivariate analysis to determine prognostic factors. All statistical tests were two‐sided, and p ≤ 0.05 was considered statistically significant. All statistical data were analyzed with GraphPad Prism 9.

## Results

### Patient characteristics

Two hundred and seventy-three newly diagnosed HIV-associated DLBCL were included in this study. The median age was 47 years (range, 21-90) at lymphoma diagnosis, and 223 patients were male (81.7%). One hundred and ninety-four (71.1%) patients were germinal center B-cell-like lymphoma (GCB) subtype. Most patients (65.2%, 178/273) had elevated lactate dehydrogenase (LDH), (83.5%, 228/273) elevated serum β_2_-microglobulin (β_2_-MG), and advanced Ann Arbor stage (78.9% 213/273) at diagnosis. High international prognostic index (IPI) score (3-5) at diagnosis was found in 65.2% (178/273) of patients. One hundred and fifty-five patients (56.8%) had extranodal involvement. Totally 53(19.4%) patients had bone marrow involvement, 24(8.8%) patients had central nervous system (CNS) involvement, 64 (23.4%) had poor Eastern Cooperative Oncology Group performance status (ECOG PS 2-4), and 150 (54.9%) had B symptoms at diagnosis. The median CD4 cell count at diagnosis was 168/μl (range, 2-1067), of whom 174 (63.7%) had a CD4 cell count below 200/μl at diagnosis. Among the Epstein-Barr virus (EBV) status, EBV load was elevated (5×10^3^ copies/ml) in 104 (38.1%) patients. Of all patients, 37(13.6%) had positive HBsAg and 7(2.6%) were positive for anti-Hepatitis C virus (HCV) antibody.

Patients were stratified based on the induction chemotherapy, where 38/273 (13.9%), 71/273 (26.0%), 73/273 (26.7%), and 91/273 (33.3%) were in the 0, 1-3, 4-6, and >6 cycles chemotherapy cohort, respectively. In the study, some patients received no anti-lymphoma therapy because of fear of discrimination and poor financial situation, some patients had less than four cycles of anti-lymphoma therapy also due to poor financial situation. This is because Central and Western China AIDS Lymphoma League (CALL) is located in southwest China, and the economic level of the patients is low. Patient characteristics were similar between the four cohorts, including gender, age, residence, educational level, HIV transmission route, cART therapy, years of HIV infection before lymphoma, ECOG-PS, the presence of B symptoms, elevated serum LDH, elevated serum β_2_-MG, Ann Arbor stage, IPI score, extra-nodal involvement, bone marrow involvement, CNS involvement, CD4 cell count, and chemotherapy regimen. The 0 cycle chemotherapy cohort featured more cases of GCB (94.7% vs 64.8% vs 68.5% vs 68.1, 0 vs 1-3 vs 4-6 vs >6, respectively, *p*=0.006). Baseline clinical characteristics are summarized in **Table 1**. Treatment cohorts were evenly balanced for demographics and disease characteristics, though the 0 cycle chemotherapy group had a higher GCB patients compared to the other cohorts (**Table 1**). In this study, there was no patient with primary HIV-associated CNS lymphoma. There was no patient along with CNS disease present at diagnosis. All 24 patients routinely administered CNS prophylaxis by intrathecal methotrexate, cytarabine and dexamethasone. A total of 20 secondary HIV-associated CNS lymphoma patients, who all had more than two extranodal sites, all died within 24 months after lymphoma diagnosis. These findings suggested very poor survival in secondary HIV-associated CNS lymphoma.

**Table 1:** Baseline clinical characteristics of patients.

Baseline characteristics	Total n=273(%)	Chemotherapy cycles				p
		0 (n=38)	1-3 (n=71)	4-6 (n=73)	>6 (n=91)	
Gender						0.095
Male	223 (81.7)	35 (92.1)	61 (85.9)	53 (72.6)	74 (81.3)	
Female	50 (18.3)	3 (7.9)	10 (14.1)	20 (27.4)	17 (18.7)	
Age, y						0.067
Median(range)	47 (21-90)	43 (21-79)	49 (21-90)	47 (23-70)	47 (22-71)	
<60	198 (72.5)	32 (84.2)	54 (76.1)	63 (86.3)	84 (92.3)	
≥60	75 (27.5)	6 (15.8)	17 (23.9)	10 (13.7)	7 (7.7)	
Residence						0.692
City/town	128 (46.9)	18 (47.4)	29 (40.8)	36 (49.3)	45 (49.5)	
Village	145 (53.1)	20 (52.6)	42 (59.2)	37 (50.7)	46 (50.5)	
Educational level						0.996
None or primary only	93 (34.1)	12 (31.6)	25 (35.2)	23 (31.5)	33 (36.3)	
Secondary education	119 (43.6)	17 (44.7)	30 (42.3)	33 (45.2)	39 (42.9)	
Tertiary education	61 (22.3)	9 (23.7)	16 (22.5)	17 (23.3)	19 (20.9)	
HIV transmission route						0.116
Heterosexual	85 (31.1)	12 (31.6)	25 (35.2)	28 (38.4)	20 (22.0)	
Homosexual	131 (48.0)	20 (52.6)	30 (42.3)	26 (35.6)	55 (60.4)	
Intravenous drug use	30 (11.0)	3 (7.9)	7 (9.9)	9 (12.3)	11 (12.1)	
Others	27 (9.9)	3 (7.9)	9 (12.7)	10 (13.7)	5 (5.5)	
ART therapy	239(87.5)	32 (84.2)	62 (87.3)	64 (87.7)	81 (89.0)	0.903
Years of HIV infection before lymphoma						0.198
Concurrent	88 (32.2)	15 (39.5)	21 (29.6)	18 (24.7)	34 (37.4)	
<3	115 (42.1)	18 (47.4)	32 (45.1)	35 (47.9)	30 (33.0)	
≥3	70(25.6)	5 (13.2)	18 (25.4)	20 (27.4)	27 (29.7)	
ECOG-PS						0.992
0-1	209 (76.6)	29 (76.3)	55 (77.5)	55 (75.3)	70 (76.9)	
2-4	64 (23.4)	9 (23.7)	16 (22.5)	18 (24.7)	21 (23.1)	
Cell-of-origin subtype						0.006
GCB	194 (71.1)	36 (94.7)	46 (64.8)	50 (68.5)	62 (68.1)	
non-GCB	79 (28.9)	2 (5.3)	25 (35.2)	23 (31.5)	29 (31.9)	
B symptoms	150 (54.9)	27 (71.1)	37 (52.1)	40 (54.8)	46 (50.5)	0.177
Elevated LDH	178 (65.2)	26 (68.4)	49 (69.0)	45 (61.6)	58 (63.7)	0.772
≥3 upper limit of normal	51 (18.7)	9 (23.7)	13 (18.3)	13 (17.8)	16 (17.6)	
Elevated β_2_-MG	228 (83.5)	30 (78.9)	61 (85.9)	60 (82.2)	77 (84.6)	0.796
Ann Arbor stage						0.108
I/II	60 (21.9)	6 (15.8)	19 (26.8)	21 (28.8)	14 (15.4)	
III/IV	213 (78.9)	32 (84.2)	52 (73.2)	52 (71.2)	77 (84.6)	
IPI						0.979
0-1	17 (6.2)	2 (5.3)	3 (4.2)	7 (9.6)	5 (5.5)	
2	78 (28.6)	10 (26.3)	21 (29.6)	22 (30.1)	25 (27.5)	
3	87 (31.9)	12 (31.6)	24 (33.8)	21 (28.8)	30 (33.0)	
4-5	91 (33.3)	14 (36.8)	23 (32.4)	23 (31.5)	31 (34.1)	
Extra-nodal	155 (56.8)	24 (63.2)	39 (54.9)	47 (64.4)	45 (49.5)	0.218
Bone marrow involvement	53 (19.4)	7 (18.4)	14 (19.7)	15 (20.5)	17 (18.7)	0.989
CNS involvement	24 (8.8)	3 (7.9)	7 (9.9)	7 (9.6)	7 (7.7)	0.953
Bulky tumor(≥7.5cm)	81 (29.7)	13 (34.2)	24 (33.8)	25 (34.2)	19 (20.9)	0.167
CD4 cell count (/μl)						0.383
Median (Range)	168 (2-1067)	120 (3-526)	169 (2-1067)	175 (12-900)	171 (5-1022)	
<50	45 (16.5)	11 (28.9)	13 (18.3)	9 (12.3)	12 (13.2)	
50-199	129 (47.3)	16 (42.1)	35 (49.3)	36 (49.3)	42 (46.2)	
≥200	99 (36.3)	11 (28.9)	23 (32.4)	28 (38.4)	37 (40.7)	
EBV(copies/ml)						0.539
<5×10^3^	169 (61.9)	25 (65.8)	46 (64.8)	40 (54.8)	58 (63.7)	
≥5×10^3^	104 (38.1)	13 (34.2)	25 (35.2)	33 (45.2)	33 (36.3)	
HBV positive	37 (13.6)	4 (10.5)	10 (14.1)	8 (11.0)	15 (16.5)	0.705
HCV positive	7 (2.6)	1 (2.6)	2 (2.8)	3 (4.1)	1 (1.1)	0.673
Chemotherapy regimen						0.202
EPOCH	80/235 (34.0)	0	24 (33.8)	25 (34.2)	21 (23.1)	
R-EPOCH	155/235 (66.0)	0	47 (66.2)	48 (65.8)	70 (76.9)	

### Treatment efficacy

In this cohort study, the median chemotherapy cycles were 4 (range, 0-19), 38 (13.9%) received no anti-lymphoma therapy, 71 (26.0%) had less than four cycles of chemotherapy, 73 (26.7%) patients received four to six cycles of chemotherapy, and 91 (33.3%) patients received more than six cycles of chemotherapy. Of the 235 patients who received chemotherapy, 80 (34.0%) received EPOCH regimen and 155 (66.0%) received R-EPOCH regimen. 239 (87.5%) were administered cART. Totally 209 patients were evaluated for best treatment response at the end of treatment, including 65 patients who received EPOCH and 144 patients who received R-EPOCH. The results indicated an overall response rate (ORR) of 63.1% and 80.6%, respectively.CR rate were 23.1% and 42.4%, respectively **(** **Table 2** **).**

**Table 2:** Response evaluation following chemotherapy in patients.

	Chemotherapy regimen	
	EPOCH	R-EPOCH
**Able to evaluate**	N=65 (%)	N=144 (%)
**Overall response rate (ORR)**	41 (63.1)	116 (80.6)
Complete response (CR)	15 (23.1)	61 (42.4)
Partial response (PR)	26 (40.0)	55 (38.2)
Stable disease (SD)	14 (21.5)	10 (6.9)
Progressive disease (PD)	10 (15.4)	18 (12.5)
**Unable to evaluate**	N=15	N=11
Ongoing treatment	15	11

### Chemotherapy cycles and patient outcome

**Figure 1:**
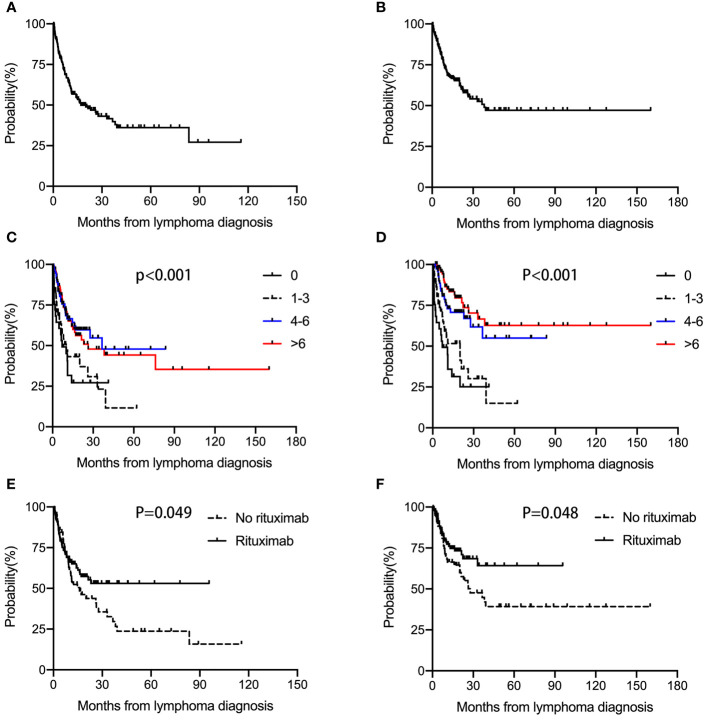
PFS and OS in HIV-associated DLBCL patients. Median PFS **(A)** and OS **(B)** were 17 months and 38.1 months, respectively. Median PFS times in the 0, 1-3, 4-6, and >6 cycles chemotherapy cohort were 7.1 months, 10.1 months, 36.5 months, and 26.4 months, respectively (p<0.001) **(C)**. Median OS times were 7.1 months, 20.0 months, not reached, and not reached, respectively (p<0.001) **(D)**. Median PFS times in the no received rituximab and received rituximab groups were 16 months and not reached (p=0.049) **(E)**. Median OS times were 27.7 months and not reached (p=0.048) **(F)**.

The median follow‐up of our cohort was 10.1 (0.1-160) months. Median PFS and OS were 17 months and 38.1 months respectively. The overall 2-year PFS and OS rates were 46.8% and 58.0%, respectively **(** **Figures 1A, B** **)** . Median PFS times in the 0, 1-3, 4-6, and >6 cycles chemotherapy cohort were 7.1 months, 10.1 months, 36.5 months, and 26.4 months, respectively (Hazard Ratio (HR)=0.713, 95% Confidence interval (CI) 0.600-0.847; *p*<0.001) **(** **Figure 1C** **)**. Median OS times were 7.1 months, 20.0 months, not reached, and not reached, respectively (HR=0.549, 95% CI 0.451-0.667; *p*<0.001) **(** **Figure 1D** **)**. These results demonstrated that 4-6 cycles chemotherapy were significantly associated with improved outcomes in HIV-associated DLBCL patients. However, >6 cycles chemotherapy did not further improve the survival of patients.

### Rituximab administration and outcomes

Median PFS times in the no received rituximab and received rituximab groups were 16 months and not reached. The overall 2-year PFS rates were 41.0% and 53.0%, respectively (*p=*0.049) **(** **Figure 1E** **)**. Median OS times were 27.7 months and not reached. The overall 2-year OS rates were 52.6% and 68.5%, respectively (*p=*0.048) **(** **Figure 1F** **)**. These data suggested that rituximab administration was significantly associated with improved outcomes.

### Prognostic factors

Univariate analysis showed that advanced stage (HR=2.172, 95% CI 1.243-3.797; *p*=0.006), high IPI score (3-5) (HR=2.452, 95% CI 1.607-3.742; *p<*0.001), B symptoms (HR=2.228, 95%CI 1.500-3.311; *p<*0.001), poor ECOG-PS (HR=1.990, 95%CI 1.357-2.919; *p<*0.001), elevated LDH (HR=1.704, 95%CI 1.145-2.535; *p<*0.001), Extra-nodal involvement (HR=1.478, 95%CI 1.013-2.155; *p*=0.043) and received less than 4 cycles chemotherapy (HR=0.713, 95%CI 0.600-0.847; *p<*0.001) were predictive of worse PFS. Age ≥60 (HR=2.346, 95%CI 1.420-3.875; *p=*0.001), high IPI score (3-5) (HR=2.960, 95% CI 1.774-4.939; *p<*0.001), B symptoms (HR=2.457, 95%CI 1.535-3.932; *p<*0.001), elevated LDH (HR=2.022, 95%CI 1.254-3.260; *p*=0.004) and received less than 4 cycles chemotherapy (HR=0.713, 95%CI 0.549-0.667; *p<*0.001) were predictive of worse OS.

Cox multivariate analysis showed that high IPI score (3-5) (HR=1.996, 95% CI 1.225-3.253; *p=*0.006), B symptoms (HR=1.623, 95%CI 1.077-2.448; *p=*0.021) and received less than 4 cycles chemotherapy (HR=0.686, 95%CI 0.575-0.819; *p<*0.001) were independent risk factors for adverse prognosis based on PFS. Age ≥60 (HR=2.207, 95%CI 1.321-3.690; *p=*0.003), high IPI score (3-5) (HR=2.926, 95% CI 1.716-4.988; *p<*0.001), B symptoms (HR=1.928, 95%CI 1.192-3.119; *p=*0.007), elevated LDH (HR=1.696, 95%CI 1.031-2.791; *p*=0.038) and received less than 4 cycles chemotherapy (HR=0.520, 95%CI 0.424-0.637; *p<*0.001) were independent risk factor for adverse prognosis based on OS **(** **Table 3** **)**.

**Table 3:** Prognostic factor analysis for progression free survival and overall survival.

Variables	Progression-free survival				Overall survival			
	Univariate		Multivariate		Univariate		Multivariate	
	HR (95% CI)	*p*	HR (95% CI)	*p*	HR (95% CI)	*p*	HR (95% CI)	*p*
Gender (F/M)	1.057 (0.670-1.667)	0.813			1.208 (0.693-2.105)	0.506		
Age (<60/≥60)	1.521 (0.938-2.466)	0.089			2.346 (1.420-3.875)	0.001	2.207 (1.321-3.690)	0.003
COO subtype (GCB/non-GCB)	1.187 (0.804-1.752)	0.388			0.823 (0.508-1.334)	0.429		
Stage (I-II/III-IV)	2.172 (1.243-3.797)	0.006	1.344 (0.743-2.431)	0.328	1.787 (0.972-3.285)	0.062		
IPI (0-2/3-5)	2.452 (1.607-3.742)	<0.001	1.996 (1.225-3.253)	0.006	2.960 (1.774-4.939)	<0.001	2.926 (1.716-4.988)	<0.001
B symptoms	2.228 (1.500-3.311)	<0.001	1.623 (1.077-2.448)	0.021	2.457 (1.535-3.932)	<0.001	1.928 (1.192-3.119)	0.007
ECOG (0-1/2-4)	1.990 (1.357-2.919)	<0.001	1.293 (0.847-1.974)	0.234	1.496 (0.946-2.366)	0.085		
CD4 (<200/≥200)	0.804 (0.546-1.184)	0.270			0.768 (0.490-1.203)	0.249		
History of HIV (year) (0/<3/≥3)	1.228 (0.984-1.531)	0.169			1.248 (0.941-1.656)	0.125		
LDH	1.704 (1.145-2.535)	0.009	1.316 (0.867-1.997)	0.197	2.022 (1.254-3.260)	0.004	1.696 (1.031-2.791)	0.038
Extra-nodal involvement	1.478 (1.013-2.155)	0.043	1.174 (0.794-1.737)	0.421	1.052 (0.689-1.607)	0.814		
Bulky tumor	0.971 (0.655-1.440)	0.885			1.084 (0.691-1.700)	0.726		
Chemotherapy cycles (0/1-3/4-6/>6)	0.713 (0.600-0.847)	<0.001	0.686 (0.575-0.819)	<0.001	0.549 (0.451-0.667)	<0.001	0.520 (0.424-0.637)	<0.001
EBV positive	1.082 (0.404-1.352)	0.562			0.923 (0.528-1.124)	0.498		

COO, Cell-of-origin subtype.

## Discussion

Due to the particularity of HIV infection, few medical centers treat HIV-associated lymphoma patients in China. In order to raise awareness of this rare group, we have set up a cooperation group, named Central and Western China AIDS Lymphoma League (CALL) in May 2021. The CALL is a public welfare organization dedicated to the diagnosis and treatment of HIV-associated lymphoma patients. This is the first multi‐center retrospective study with HIV-associated DLBCL in eleven China academic centers from CALL from October 2008 to October 2021. The present study included 273 HIV-associated DLBCL cases, which is the largest cohort reported in China to date. To the best of our knowledge, this is also the first systematic study to explore the effect of chemotherapy cycles on prognosis in a large series of HIV-associated DLBCL.

HIV-associated malignancies are divided into acquired immune deficiency syndrome-defining and non-acquired immune deficiency syndrome-defining cancers based on the coincidence rate. Kaposi’s sarcoma, Non-Hodgkin’s lymphoma and invasive cervical cancer are considered acquired immune deficiency syndrome- defining cancers. Other cancers, which including Hodgkin’s lymphoma, hepatocellular carcinoma, oral and pharyngeal cancers, lung cancer, anal cancer, vulvar cancer and penile cancer, are considered non-acquired immune deficiency syndrome-defining cancers (1, 2). The latest research show that HIV-associated Non-Hodgkin’s lymphoma is the highest incidence rate among HIV-associated malignancies in the United States (2). DLBCL is the most common subtypes of HIV-associated lymphoma (3).

The present cohort study showed that the median age was 47 years (range, 21-90) at lymphoma diagnosis, and 223 patients were male (81.7%), in agreement with the overall male-to-female HIV incidence rate in China (9). For lymphoma patients, who no infection HIV, most have painless lymphadenopathy as the primary presentation (10). Compared with HIV-negative lymphoma, the initial symptoms of HIV-associated lymphoma are more variable, with more being advanced stage at diagnosis, often accompanied by B-symptoms and showing more manifestations of extranodal involvement (11–14). The present study showed that most patients had advanced Ann Arbor stage (78.9% 213/273), (65.2%, 178/273) elevated LDH, (56.8%, 155/273) extranodal involvement and (65.2%, 178/273) High IPI score (3-5). These results suggest that HIV-associated DLBCL is more aggressive.

Currently, there are no standard guidelines for treatment of HIV-associated DLBCL. In the pre-cART era, median survival times only 5 to 8 months for HIV-associated DLBCL patients. This is because patients are easily complicated by opportunistic infections, resulting in an increase in mortality (4, 5). In the cART era, combining cART with intensity chemotherapy could significantly improve survival in HIV-associated DLBCL patients, with 5-year OS up to 55% (15).

Rituximab can significantly improves the survival of HIV-negative DLBCL patients (16). In the cART era, Coutinho showed that HIV-associated DLBCL receiving rituximab have better OS (94% vs 77%, *p*=0.03) and PFS (78% vs 64%, *p*=0.03) compared with HIV-negative patients (17). Our previous single-center study showed that HIV-associated DLBCL receiving rituximab have better median PFS (not reached vs 12 months, *p=*0.006) and median OS (not reached vs 36 months, *p=*0.021) compared with no receiving rituximab (8). In this CALL study, the overall 2-year PFS rates in the no receiving rituximab and receiving rituximab groups were 41.0% and 53.0%, respectively (*p=*0.049). The overall 2-year OS rates were 52.6% and 68.5%, respectively (*p=*0.048). The current data also suggested that rituximab administration was significantly associated with improved outcomes in HIV-associated DLBCL. In AMC034, rituximab combined with EPOCH was assessed, and the results were compared with R-CHOP in AMC010. The results showed that a higher CR rate (69%) was obtained for R-EPOCH compared with R-CHOP (47%) (18). In the CALL study, totally 209 patients were evaluated for best treatment response at the end of treatment, including 65 patients who received EPOCH and 144 patients who received R-EPOCH. The results indicated an overall response rate (ORR) of 63.1% and 80.6%, respectively. Complete response (CR) rate were 23.1% and 42.4%, respectively. Further AMC075 study found that survival in HIV-associated DLBCL is not significantly improved after R-EPOCH combination with vorinostat, an histone deacetylase (HDAC) inhibitor (7). So far, R-EPOCH is more helpful in prolonging survival in patients with HIV-associated DLBCL. Whether HIV infection is a risk factor for increased mortality in HIV-associated lymphoma remains highly controversial. However, in DLBCL, HIV infection is no longer an independent predictor of death after controlling for mixed factors such as the frequency of rituximab use (19).

The optimal treatment cycles option of first line induction chemotherapy for HIV-associated DLBCL remains undefined. Compared with HIV-negative DLBCL patients, HIV-associated DLBCL patients are difficult to accept long-term hospitalization chemotherapy, whether there is an appropriate reduction in the number of chemotherapy cycles, the survival of patients will not be affected? Wu D retrospectively analyzed the clinical features of 100 HIV-associated lymphoma patients, which including 66 HIV-associated DLBCL. 15 patients (15%) did not received chemotherapy. Compared to those who did not received chemotherapy, the 2-year OS rate (41.0% vs. 0%, *p*<0.001), and CR rate (20.0% vs. 0%, *p*=0.037) were significantly higher in patients who received chemotherapy (20). Another Chinese study also found that more than 1/5 (24%, 25/104) patients with HIV-associated DLBCL did not receive chemotherapy (21). In our previous single-center study, of all 86 HIV-associated aggressive B-cell lymphoma patients, 22 (25.6%) did not receive chemotherapy because of fear of discrimination and poor financial situation, including 16 DLBCL patients (8). Once the chemotherapy is given up, the prognosis of the patients will become very poor (8, 20–24). Based on this, we carried out this study. To the best of our knowledge, this is also the first systematic study to explore the effect of chemotherapy cycles on prognosis in a large series of HIV-associated DLBCL. In the CALL study, cox multivariate analysis showed that received less than 4 cycles chemotherapy (HR=0.520, 95%CI 0.424-0.637; *p<*0.001) were independent risk factor for adverse prognosis.

## Conclusion

In summary, our multi-center study suggested that received less than 4 cycles chemotherapy predicted poor OS and PFS in HIV-associated DLBCL patients. Therefore, we recommend that patients receive at least 4 cycles of systemic chemotherapy. More high-quality randomized controlled studies were needed to test our findings.

## Data availability statement

The raw data supporting the conclusions of this article will be made available by the authors, without undue reservation.

## Ethics statement

The studies involving human participants were reviewed and approved by Institutional Review Board. The patients/participants provided their written informed consent to participate in this study.

## Author contributions

CW, YW, JL, and HM conceived and designed the study, analyzed the data, and drafted and revised the paper. HZ, YC, and YL conceptualized and designed the study. All authors provided critical comments to the manuscript. All authors contributed to the article and approved the submitted version.

## References

[1] Wang CY Liu J Liu Y. Progress in the treatment of HIV-associated lymphoma when combined with the antiretroviral therapies. Front Oncol (2022) 11:798008. doi: 10.3389/fonc.2021.798008 35096597 PMC8792758

[2] Yarchoan R Uldrick TS. HIV-Associated cancers and related diseases. N Engl J Med (2018) 378(22):1029–41. doi: 10.1056/NEJMra1615896 PMC6890231 29539283

[3] Simard EP Pfeiffer RM Engels EA. Cumulative incidence of cancer among individuals with acquired immunodeficiency syndrome in the united states. Cancer (2011) 117(5):1089–96. doi: 10.1002/cncr.25547 PMC3052856 20960504

[4] Gates AE Kaplan LD. Biology and management of AIDS-associated non-hodgkin’s lymphoma. Hematol Oncol Clin North Am (2003) 17:821–41. doi: 10.1016/S0889-8588(03)00041-8 12852657

[5] Kaplan LD Straus DJ Testa MA. Low-dose vs standard dose m-BACOD chemotherapy for non-hodgkin’s lymphoma associated with human immunodefificiency virus infection. N Engl J Med (1997) 336:1641–8. doi: 10.1056/NEJM199706053362304 9171066

[6] Barta SK Lee JY Kaplan LD Noy A Sparano JA. Pooled analysis of AIDS malignancy consortium trials evaluating rituximab plus CHOP or infusional EPOCH chemotherapy in HIV-associated non-Hodgkin lymphoma. Cancer (2012) 118(16):3977–83. doi: 10.1002/cncr.26723 PMC3310940 22180164

[7] Ramos JC Sparano JA Chadburn A Reid EG Ambinder RF Siegel ER. Impact of myc in HIV-associated non-Hodgkin lymphomas treated with EPOCH and outcomes with vorinostat (AMC-075 trial). Blood (2020) 136(11):1284–97. doi: 10.1182/blood.2019003959 PMC7483436 32430507

[8] Wang C Liu J Lei H Li Y Wu J Guo B. Clinical characteristics and outcomes of newly diagnosed patients with HIV-associated aggressive b-cell NHL in China. J Cell Mol Med (2022) 26(19):5067–77. doi: 10.1111/jcmm.17534 PMC9549495 36056692

[9] Cao W Hsieh E LiT S. Optimizing treatment for adults with HIV/AIDS in china: Successes over two decades and remaining challenges. Curr HIV/AIDS Rep (2020) 17(1):26–34. doi: 10.1007/s11904-019-00478-x 31939111 PMC6989417

[10] Flepisi BT Bouic P Sissolak G Rosenkranz B. Biomarkers of HIV-associated cancer. Biomarkers Cancer (2014) 6:11–20. doi: 10.4137/BIC.S15056 PMC4085100 25057241

[11] Gabarre J Raphael M Lepage E Martin A Oksenhendler E Xerri L. Human imunodeficiency virus-related lymphoma: Relation between clinical features and histologic subtypes. Am J Med (2001) 111:704–11. doi: 10.1016/S0002-9343(01)01020-8 11747850

[12] Riedel DJ Rositch AF Redfield RR Blattner WA. HIV-Associated lymphoma sub-type distribution immunophenotypes and survival in an urban clinic population. Leuk Lymphoma (2016) 57(2):306–12. doi: 10.3109/10428194.2015.1055483 26025299

[13] Rudresha AH Khandare PA Lokanatha D Linu AJ Suresh Babu MC Lokesh KN. HIV/AIDS-related lymphoma: Perspective from a regional cancer center in India. Blood Res (2019) 54(3):181–8. doi: 10.5045/br.2019.54.3.181 PMC6779940 31730692

[14] Atallah-Yunes SA Murphy DJ Noy A. HIV-Associated burkitt lymphoma. Lancet Haematol (2020) 7(8):e594–600. doi: 10.1016/S2352-3026(20)30126-5 32735838

[15] Collaboration of Observational HIV Epidemiological Research Europe ( COHERE) study group. Prognosis of HIV-associated non-Hodgkin lymphoma in patients starting combination antiretroviral therapy. AIDS (2009) 23(15):2029–37. doi: 10.1097/QAD.0b013e32832e531c 19531926

[16] Pfreundschuh M Trumper L Osterborg A Pettengell R Trneny M Imrie K. CHOP-like chemotherapy plus rituximab versus CHOP-like chemotherapy alone in young patients with good-prognosis diffuse large-b-cell lymphoma: A randomised controlled trial by the MabThera international trial (MInT) group. Lancet Oncol (2006) 7:379–91. doi: 10.1016/S1470-2045(06)70664-7 16648042

[17] Coutinho R Pria AD Gandhi S Bailey K Fields P Cwynarski K. HIV Status does not impair the outcome of patients diagnosed with diffuse large b-cell lymphoma treated with r-CHOP in the cART era. AIDS (2014) 28(5):689–97. doi: 10.1097/QAD.0000000000000133 24418826

[18] Sparano JA Lee JY Kaplan LD Levine AM Ramos JC Ambinder RF. Rituximab plus concurrent infusional EPOCH chemotherapy is highly effective in HIV-associated b-cell non-Hodgkin lymphoma. Blood (2010) 115(15):3008–16. doi: 10.1182/blood-2009-08-231613 PMC2858478 20023215

[19] Cingolani A Cozzi Lepri A Teofili L Galli L Mazzotta V Baldin GM. Survival and predictors of death in people with HIV-associated lymphoma compared to those with a diagnosis of lymphoma in general population. PloS One (2017) 12(10):e0186549. doi: 10.1371/journal.pone.0186549 29088223 PMC5663375

[20] Wu D Chen C Zhang M Li Z Wang S Shi J. The clinical features and prognosis of 100 AIDS-related lymphoma cases. Sci Rep (2019) 9(1):5381. doi: 10.1038/s41598-019-41869-9 30926889 PMC6441082

[21] Wu J Miao Y Qian C Tao P Wang X Dong X. Clinical characteristics and outcomes in HIV-associated diffuse large b-cell lymphoma in China: A retrospective single-center study. J Cancer (2021) 12(10):2903–11. doi: 10.7150/jca.51027 PMC8040883 33854591

[22] Carbone A Vaccher E Gloghini A. Hematologic cancers in individuals infected by HIV. Blood (2022) 139(7):995–1012. doi: 10.1182/blood.2020005469 34469512

[23] Hou JZ Ye JC Pu JJ. Novel agents and regimens for hematological malignancies: Recent updates from 2020 ASH annual meeting. J Hematol Oncol (2021) 14(1):66. doi: 10.1186/s13045-021-01077-3 33879198 PMC8059303

[24] Wang L Li LR Young KH. New agents and regimens for diffuse large b cell lymphoma. J Hematol Oncol (2020) 13(1):175. doi: 10.1186/s13045-020-01011-z 33317571 PMC7734862

